# The Effect of Polarized Training on the Athletic Performance of Male and Female Cross-Country Skiers during the General Preparation Period

**DOI:** 10.3390/healthcare9070851

**Published:** 2021-07-06

**Authors:** Tae Ho Kim, Joung Kyue Han, Ji Young Lee, Yong Chul Choi

**Affiliations:** 1 Department of Health Science, Sports for All, Jangan University, Hwaseong 18331, Korea; thgy1125@jangan.ac.kr; 2 College of Sports Science, Chung-ang University, Anseong 17546, Korea; jkhan@cau.ac.kr; 3 Department of Physical Education, Gangneung-Wonju National University, Gangneung 25457, Korea

**Keywords:** polarized training, anaerobic threshold, endurance fitness, cross-country ski

## Abstract

This study aimed to analyze the effect of 12 weeks of polarized training on body composition, cardiorespiratory function, and upper-body power of male and female cross-country skiers during the general preparation period. A total of 16 national cross-country skiers (8 male and 8 female; 8 national cross-country skiers and 8 national biathlon athletes) participated. Polarization training was conducted for 12 weeks from May to July in 2019 during the general preparation period for cross-country skiers. The low-weight, high-repetition method was used for strength training. The effect of the polarized training on body composition, maximum oxygen intake (VO_2_max), respiratory exchange rate, all-out time, and ski ergometer exercise time was assessed. There was no change in weight, BMI, and muscle mass in male and female cross-country skiers following the 12 weeks of polarized training (*p* > 0.05). Male body fat percentage (pre 18.1%, post 12.7%) and female body fat percentage (pre 29.1%, post 21.4%) showed a significant decrease (*p* < 0.05). After training, VO_2_max increased by 7.72% in male athletes (pre 71.05 mL/kg/min, post 77.0 mL/kg/min) and 6.32% in female athletes (pre 60.26 mL/kg/min, post 64.33 mL/kg/min). Treadmill exercise time increased by 5.39% for male athletes (pre 1038 s, post 1064 s) and 2.23% for female athletes (pre 855 s, post 874 s). However, there was no significant difference between male and female athletes (*p* > 0.05). The 50% recovery time from the maximum heart rate to the target heart rate decreased by 64.52% in males (pre 168.8 s, post 102.6 s) and 6.48% in females (pre 135 s, post 129.6 s). Significant differences were found only in male athletes (*p* < 0.05). The double-pole 500 m exercise duration for the ski ergometer significantly decreased after the training for both sexes (*p* < 0.05). In this study, the 12 weeks of polarized training improved the body composition and athletic performance of all cross-country skiers. Interestingly, in this study, we confirmed that polarized training had a better effect on cardiorespiratory function in male cross-country skiers than in female cross-country skiers. Conversely, we found that the outcomes of the ski ergometer exercise factors were more effective in female athletes than in male athletes. Therefore, we insist that when applying a polarized training program to athletes, it should be planned in detail by sex, exercise amount, intensity, and type of training.

## 1. Introduction

Cross-country skiing is a sport that requires endurance, as with cycling and running [1,2,3]. Generally, in endurance sports, the higher the performance, the better the maximum oxygen intake. In cross-country skiing events, world-class athletes are reported to have higher maximum oxygen intake than the national-level athletes [4,5,6]. In addition, compared to national-level cross-country skiers, world-class cross-country skiers have superior anaerobic power, muscular endurance and muscle power, and high aerobic and endurance capabilities [1,7,8,9,10,11,12].

Therefore, endurance training is very important for cross-country skiers because it improves capillary density, myoglobin content, and mitochondria number and size, which improve maximum oxygen intake, aerobic metabolism, and energy production [13,14,15,16]. An important consideration in endurance training is the efficient distribution of exercise intensity, duration, and frequency. Typical endurance training methods including prolonged high-volume, low-intensity exercise, lactate threshold training, low-volume, high-intensity interval training, and polarized (POL) training combine low intensity, high momentum, and low-to-moderate high interval training [17,18,19,20]. Successful cross-country skiers use the POL training model to perform endurance training in 85–90% of their annual exercise, and perform longer low-intensity endurance training compared to national skiers [2,6,13,21]. In addition, it is more effective to perform strength training together with endurance training than endurance training only for short-term endurance exercise performance [15,22,23,24].

All sports events have a training cycle to achieve the best athletic ability in important competitions [25]. Cross-country skiing is also considered to have seasonal characteristics: general preparation period 1 (May to July), general preparation period 2 (August to October), specific preparation period (November to December), and the competition period (January to March) [21,23]. It is important to plan the training program according to the purpose of the training cycle and the individuality of the trainees. The purpose of the general preparation period is to improve the athlete’s general fitness level and work capacity and to maximize adaptability in preparation for a high level of training. In addition, excessive high-intensity training and a large volume of training during the preparation period can cause muscle injury and overtraining; therefore, it is necessary to plan appropriate and detailed scientific training [26,27,28,29,30].

There is a lack of research on the correlation between the training cycle and POL training in cross-country skiing. Therefore, this study aimed to plan an annual program for cross-country skiers by analyzing the effects of POL training on body composition, cardiopulmonary function, and the upper-body power of male and female cross-country skiers during the general preparation period (12 weeks) in order to provide basic information about the preparation period.

Additionally, we expected that POL training would have a positive effect on cardiorespiratory function in both male and female athletes. However, we also predicted that exercise time and upper-body strength power would differ due to differences in skeletal muscle mass and body fat percentage.

## 2. Methods

### 2.1. Participants

Sixteen (8 male, 8 female) cross-country skiers from the Korean national team (cross-country ski and biathlon) participated in this study. This study procedure and informed consent document were approved and reviewed by the Ethics Committee of Gangeung-Wonju National University, Korea (GWNUIRB-2021-11; approval date: 25 February 2021). This research was performed in accordance with the Declaration of Helsinki principles. The physical characteristics of the participants are presented in Table 1.

### 2.2. Design and Procedures

This experiment was conducted from May to July in 2019 for 12 weeks. To analyze the effect of POL training on the exercise performance of cross-country skiers in the general preparation period, we compared and analyzed the following exercise performance factors before and after the training period: the all-out time for body composition, cardiopulmonary function, exercise load test, and the ski ergometer exercise time.

### 2.3. Anthropometric Measurements and Body Composition

Participants’ height and body weight were respectively assessed to proximate 0.1 cm and 0.1 kg. Also, body mass index (BMI) was measured according to the following formula: kg/m^2^. Body composition such as body fat and lean mass, and body fat percentage (%), were measured using bioelectrical impedance analysis (InBody 3.0, Inbody, Korea). Participants’ body composition was measured before training and after 12 weeks of POL training. For accurate measurement, measurements were taken without nutrient intake for more than 10 h.

### 2.4. Graded Exercise Test

The maximum exercise test was carried out using respiratory gas separation equipment: Quark CPET (Cosmed Co., Rome, Italy). In addition, the Bruce protocol was used. Exercise tests were conducted until the participants were all-out, and the endpoint was marked by a participant’s RPE scale of 17 or more and a respiratory ratio of over 1.15. Additionally, for the safety of athletes, blood pressure and ECG (electrocardiogram) were continuously monitored during the graded exercise test. However, no abnormal ECG rhythms were observed in the athletes participating in the experiment [31,32,33].

### 2.5. Ski Ergometer Test

To evaluate the participants’ exercise time through upper-body muscle power, a maximum upper-body power strength exercise test was performed using a Ski Ergometer (Concept II, Morrisville, NC, USA) [34,35,36]. Strokes were performed for a total distance of 500 m using the double-poling technique, and the duration was automatically recorded on the ergometer monitor. Three sets of the tests were evaluated before and after the POL training program with 2 min of rest per set.

### 2.6. Training Program

POL training utilized the training program suggested for existing cross-country athletes [13,17,19,37]. The Polarized training method is a training method that performs 70–80% of the total amount of exercise at low intensity (HRmax 65–80%), 5–10% at middle intensity (HRmax 80~88%), and the remaining 15–20% at high intensity (HRmax 88~100%). It is the training method that achieves possibly the greatest improvement in the key variables related to the endurance performance of athletes [17,38]. For strength training, low weight and high repetitions were performed, focusing on the tissue adaptation period which accounts for 18% of the total training volume. The detailed training program is shown in Table 2.

### 2.7. Statistical Analysis

All statistical analyses were conducted using SPSS Version 20.0 (IBM, Armonk, NY, USA) for Windows. To analyze the effect of the 12 weeks of POL training on body composition, maximum oxygen intake (VO_2_max), respiratory exchange rate (RER), all-out time, and ski ergometer exercise time, we performed a paired sample *t*-test. In addition, the differences between men and women athletes were compared using a covariance analysis of the Wilcoxon signed rank test. All statistically significant levels were set at *p* < 0.05.

## 3. Results

Female cross-country skiers showed statistically significant decreases in weight, BMI, and body fat percentage after 12 weeks of POL training (*p* < 0.05). There was no statistically significant difference in the body weight and BMI of female skiers, but body fat percentage decreased significantly after the POL training (*p* < 0.05). However, for other variables we did not find any significant differences between male and female athletes (*p* > 0.05).

The maximum oxygen intake increased by 8.37% for male athletes and 6.75% for female athletes after the training, but no statistically significant difference was observed (*p* > 0.05). After the training, treadmill exercise time increased by 5.39% for male athletes and 2.23% for female athletes. However, there was no significant difference between male and female athletes (*p* > 0.05). The recovery time from the maximum heart rate to the target heart rate of 50% decreased by 64.52% in males, with a statistically significant difference (*p* < 0.05); but it decreased by 6.48% in females, with no statistically significant difference (*p* > 0.05). Excluding ski ergometer 2 set time, the ski ergometer exercise time significantly decreased after the training for both males and females (*p* < 0.05). The above results are shown in Table 3.

Meanwhile, we compared all variables and results of male and female athletes using ANCOVA analysis. As a result, after 12 weeks of POL training, it was confirmed that there was a difference between male and female athletes in weight, BMI, body fat percentage, oxygen intake and maximum oxygen intake, and aerobic threshold (*p* > 0.05). However, for other variables, we could not confirm differences between male and female athletes. The above results are shown in Table 4.

## 4. Discussion

In general, it is desirable to establish a control group in order to confirm the effect of training and reduce experimental error. The control group should be provided the equal environmental condition as the experimental group. All participants in this study were trained through the national training center’s standardized POL training program; it was almost impossible to find a control group with identical environmental conditions. Therefore, in this study we also tried to compensate for the shortcomings of this study regarding the absence of a control group by comparing male and female athletes.

POL training is an intervention method to improve the performance of endurance athletes [38,39]. In this study, it was confirmed that POL training improved the body weight, body mass index, and body fat percentage of female skiers and influenced the changes in body fat percentage of male skiers. Body composition is the most important factor for health-related physical strength and has a high correlation with the performance of cross-country athletes [40,41,42]. High weight and body fat percentage negatively affect physical strength factors such as agility and quickness [43]. It is reported that it is important to maintain as much muscle mass and as little body fat percentage as possible in order to have a positive effect on athletes’ performance [44]. In a study that analyzed the body composition of athletes of the US Ski Team, the average body fat percentage was 6.1% in males and 13.1% in females, and high weight and body fat percentage negatively affected physical strength factors such as agility and quickness [19]. Therefore, it is important for athletes to maintain as much muscle mass as possible and as little body fat as possible in order to observe a positive effect on their performance [23]. It is a common phenomenon that when weight and body fat percentage decrease, muscle mass also decreases. In our study, despite the decrease in body weight and body fat percentage, there was no statistically significant change in muscle mass, which was similar to previous studies that showed the effectiveness of POL training in reducing body fat percentage and increasing muscle mass [7,12,22]. However, in most POL training studies, those that classified the training cycles were insufficient. The results of this study suggest that POL training is advantageous in maintaining the muscle mass and effectively controlling the body fat percentage of cross-country skiers during the general preparation period. All participants in this study were provided with the same diet by a nutritionist at the national training center.

Maximum oxygen intake is a measure of an athlete’s cardiopulmonary function and is one of the indicators that has a significant impact on sports that require aerobic capacity [45,46,47]. According to a previous study, the maximum oxygen intake levels for male and female cross-country skiers worldwide are 90–95 and 73–79 mL/kg/min, and VO_2_ is 5.5 to 6.5 L/min for men athletes and 4.0 to 5.0 L/min for women athletes. These results suggest that the participants’ lower VO_2_max was slower than that of world-class athletes. [11,48]. The results of this study showed that there was a slight increase in the VO_2_max in both males and females after POL training for 12 weeks. These results suggest that POL training can be an important intervention in improving cardiorespiratory function in both male and female skiers during general preparation. According to the training cycle, elite sports athletes can change the training volume, training intensity, and training type to improve their performance [49]. Meanwhile, some studies suggested that VO_2_max could be improved by increasing the ratio of HIIT(High Intensity Interval Training) and LT(Lactate Threshold) training in POL until the competition period [50,51]. Generally, the increase rate of VO_2_max for female athletes was lower than that for male athletes. Therefore, it is necessary to increase the ratio of HIIT and LT training to female athletes when planning POL training during the general preparation period.

Heart rate is a very useful index for predicting the energy system. Also, it is the most basic variable that indicates the activity of exercise intensity and physical stress. Coaches of endurance sports such as cross-country skiing, marathon, and cycling use the monitoring of heart rate to plan training programs [27,52,53]. During exercise, maximal oxygen uptake and maximal heart rate form a proportional relationship. However, athletes with higher cardiorespiratory capacity have higher VO_2_max and lower heart rates at the same load intensity. It is known that when cardiac output increases, the amount of oxygen to the tissues increases and thus the heart rate decreases. It has been reported that an increase in VO_2_max decreases heart rate, which improves exercise records. However, many studies showed that well-trained elite athletes do not experience significant changes in maximum heart rate during high-intensity training [52,54,55,56,57]. The steeper the slope, the more the skier’s heart rate increases to the level of his maximum heart rate. Therefore, it is important to reduce an athlete’s heart rate quickly on flat ground and downhill to win the race [58,59]. Although no change in maximum heart rate was observed in our study, we found that heart rate recovered faster in male athletes than in female athletes. Heart rate is related to cardiac output. We suggest that POL training may reduce recovered time to heart rate through the VO_2_max increase in male athletes. In addition, we confirmed that the exercise time increased along with the VO_2_max of male and female athletes after the POL training program.

In addition to the cardiorespiratory function, the important fitness factors for Nordic skiers are muscle power and endurance [1,60]. A cross-country skier’s upper-body strength is one of the important factors for mass start and sprint race performance. Strong upper-body strength is associated with a lower heart rate and has physiological benefits. The ski ergometer not only evaluates cardiorespiratory function, muscle fatigue, and performance, but also improves upper-body strength [5,23,61]. The ski ergometer test has two methods of evaluating arrival time and distance. Shorter time and longer distances can be seen as an improvement in upper-body power [62,63]. We assessed the upper-body power in male and female athletes using a ski ergometer. As a result of analyzing the effects of POL training and low-weight, high-repetition weight training on the upper-body power of male and female athletes, it was found that both groups of male and female athletes increased speed. In particular, it was found to increase the upper-body power of female athletes more than male athletes. The results of this study can be interpreted as the same as those of a study showing that the physiological adaptability of female athletes was greater than male athletes as a result of ski ergometer training for 33 elite skiers [36]. The POL training and low-weight, high-repetition weight training program applied in this study can be suggested as an effective training program for female athletes in the general preparation period. However, in order to improve the upper-body power of male athletes, it is considered that it is necessary to increase the total weight of the strength program or to perform additional strength training.

Results from this study shows that 12 weeks of POL training positively affect the general quasi-term of cross-country skiers, as shown in previous studies [3,7,16] that reported that the POL training program improves the body composition and physical strength of athletes. To the best of our knowledge, this study is the first to report on the effectiveness of POL training in Asian women athletes.

On the other hand, in this study, to compare the differences between male and female athletes regarding the effects of POL training after 16 weeks of POL training, pre-measured values for various variables for male and female cross-country skiers were covariance-processed, and then the difference between before and after each variable was analyzed. It was possible to confirm the difference between male and female athletes in changes in body weight, body mass index, and body fat percentage. Furthermore, variations in the body weight and BMI of female cross-country skiers were slightly larger than those of male skiers, and there were no variation differences in muscle mass between male and female cross-country skiers. The body fat percentage of male cross-country skiers significantly decreased after the training period compared to female cross-country skiers. These results suggest that POL training is helpful in improving and maintaining the body composition of both male and female athletes; but they also suggest that men cross-country skiers’ body fat percentage can be significantly reduced to improve athletic performance. Results that disprove these findings exist in our study results, and it was confirmed that the recovery time (heart rate beat) and VO_2_max values—the most representative variables that measure cardiopulmonary function—improved significantly in male athletes after the training period compared to female athletes. This implies that POL training can improve the cardiopulmonary function of male cross-country skiers more than that of female cross-country skiers. Interestingly, in this study, we found that female athletes were more effective than male athletes in ski ergometer exercises that assessed upper-body strength and muscular endurance. Therefore, we suggest that it is necessary to apply the training program differently for male and female athletes when planning the POL training program during the general preparation period. This study suggests that polarized training will help in the planning of training programs for women and men athletes. This study has one limitation. The sample size used in this study is very small, which is due to the limited number of Korean national cross country skiers above the age of 20 years.

## 5. Conclusions

From this study we confirmed that 12 weeks of polarized training had a positive effect on the body composition of male and female cross-country skiers. In addition, it was confirmed that it contributed to the improvement in the athletic performance of cross-country skiers by improvement of cardiorespiratory function and upper-body strength. Furthermore, our study found that polarized training had a better effect on cardiorespiratory function in male cross-country skiers than in female cross-country skiers. The ski ergometer exercise, which evaluates upper-body strength and muscular endurance, was found to be more effective for female athletes than male athletes. Therefore, to implement the polarized training program, it is necessary to separate male and female athletes and plan in detail the amount of training, training intensity, and type of training required.

## Figures and Tables

**Table 1:** Baseline demographic characteristics of the men and women cross-country skiers.

Sex (n)	Age (yrs)	Height (cm)	Weight (kg)	Body Fat (%)	Muscle Mass (kg)
Male (8)	22.7 ± 3.4	171.5 ± 6.6	72.2 ± 5.19	18.1 ± 7.12	34.1 ± 2.68
Female (8)	24.1 ± 4.3	162 ± 6.0	57.3 ± 5.96	25.2 ± 4.3	29.1 ± 7.7

Data are presented as mean ± standard deviation.

**Table 2:** Training Program.

Day	Variables	A.M. Training			P.M. Training			
Mon	Event	Roller Classical			Trekking Run			
	Time	40-22-30 min speed 10 s (ex)/120 s (re) × 10 set			90 min			
	Intensity	zone 2-5-1			zone 1			
Tue	Event	Roller Skate			Weight Training + Core Training			
	Time	40-20-40 min			90 min			
	Intensity	zone 2-3-1			65%(1RM) × 15rd × 3 set recovery time 90 s			
Wed	Event	Mountain Pole Walk			REST			
	Time	180 min						
	Intensity	zone 1						
Thu	Event	Roller Classical			OTHER SPORTS (fun sports)			
	Time	90 min			90 min			
	Intensity	zone 2			FREE			
Fri	Event	Roller Skate			Weight Training + Core Training			
	Time	40-40-30 min (5 min(ex)/3 min(re) × 5 set)			90 min			
	Intensity	zone 2-4-1			65%(1RM) × 15rd × 3 set recovery time 90 s			
Sat	Event	BIKE			Rest			
	Time	180 min						
	Intensity	zone 1						
Sun	Event	Rest			RUN			
	Time				60 min			
	Intensity				zone 1			
Intensity	zone 1	zone 2	zone 3	zone 4	zone 5	Weight training	Other sports	Total
	Low intensity		Middle intensity	High intensity				
Heart rate (%max)	60–72	72–82	82–88	88–92	92–100			
Time	590	230	20	40	22	180	90	1172
Training as a % total volume	50.34	19.62	1.70	3.41	1.87	15.35	7.67	100
Section	4	1	1	1	1	2	1	11
Weight event	65%(1RM) × 15rd × 3 set, recovery time 90 s Bench press (put legs on the bench, not on the floor) & pull-down Triceps pull-downs, deadlift Rowing/arm-pull while sitting & arm-press with dumb-bells using incline bench, legs: squats & hamstring curl, single-leg squat, side squat							
Core training	Exercise 3 set, recovery 60 s Supine plank, prone plank, side plank, side plank-leg motions statically, spine in a neutral position, Swiss ball training(inclined press-ups, top position, single-leg holds, quadruped motions)							

Abbreviation: 1RM, one repetition maximum; A.M. training, ante meridiem; P.M. training, post meridiem.

**Table 3:** Changes in anthropometrics, graded exercise test variables, ski ergometer variables between pre- and post-exercise training program.

Variables	Male			Female		
	Pre	Post	*p*-Value	Pre	Post	*p*-Value
Weight (kg)	72.2 ± 5.19	68.8 ± 4.08	0.079	57.3 ± 5.96	51.1 ± 3.90	0.120
BMI (kg/m^2^)	24.6 ± 2.10	23.4 ± 1.10	0.058	21.9 ± 2.71	19.4 ± 3.90	0.120
Muscle mass (kg)	34.1 ± 2.68	32.6 ± 5.40	0.916	29.1 ± 7.7	29.1 ± 7.7	0.263
Body fat (%)	18.1 ± 7.12	12.7 ± 2.74	0.028 *	25.2 ± 4.3	21.4 ± 4.2	0.013 *
VO_2_max (L min^−1^)	4.95 ± 0.39	5.17 ± 0.14	0.069	3.16 ± 0.39	3.28 ± 0.16	0.069
VO_2_max (mL/min^−1^/kg^−1^)	71.05 ± 7.90	77.0 ± 4.49	0.327	60.26 ± 4.20	64.33 ± 3.29	0.233
HRmax (beat/min)	192 ± 9	192 ± 6	0.833	192 ± 9	193 ± 11	0.612
AT (L/min)	3.02 ± 0.84	3.29 ± 0.90	0.499	2.18 ± 0.35	2.28 ± 0.21	0.575
ATHR (beat/min)	155 ± 20	155 ± 15	0.735	165 ± 9	170 ± 11.72	0.121
All-out time (s)	1038 ± 46.1	1064 ± 49.6	0.161	855.2 ± 53.4	874.75 ± 52.6	0.120
Recovery time (s)	168.8 ± 56.1	102.6 ± 22.9	0.036 *	138 ± 48.8	129.6 ± 56.0	0.327
Ski ergometer						
1 set time (s)	122.5 ± 6.5	117 ± 2.4	0.028 *	149.3 ± 20.8	142.6 ± 8.36	0.263
2 set time (s)	123.4 ± 8.7	121.9 ± 9.5	0.400	155.3 ± 13.1	144.7 ± 8.71	0.021 *
3 set time (s)	126.1 ± 7.0	123.6 ± 8.9	0.233	150.3 ± 9.73	144.7 ± 8.46	0.021 *

* p < 0.05; data are presented as the mean ± standard deviation. *p*-values were obtained by Wilcoxon signed rank test. Abbreviation: BMI, body mass index; VO_2_max, maximal oxygen uptake; HRmax, heart rate maximum; AT, anaerobic threshold; ATHR, anaerobic threshold heart rate.

**Table 4:** Comparison of anthropometrics, graded exercise test variables, ski ergometer variables between pre- and post-exercise training program in skiers.

Variables	Group	Pre	Post	F	*p*-Value
Weight (kg)	Male	72.2 ± 5.19	68.8 ± 4.08	17.159	0.003 *
	Female	57.3 ± 5.96	51.1 ± 3.90		
BMI (kg/m^2^)	Male	24.6 ± 2.10	23.4 ± 1.10	26.487	<0.001 *
	Female	21.9 ± 2.71	19.4 ± 3.90		
Muscle mass (kg)	Male	34.1 ± 2.68	32.6 ± 5.40	0.456	0.511
	Female	29.1 ± 7.7	29.1 ± 7.7		
Body fat (%)	Male	18.1 ± 7.12	12.7 ± 2.74	12.537	0.004 *
	Female	25.2 ± 4.3	21.4 ± 4.2		
VO_2_ (mL/kg/min)	Male	4.95 ± 0.39	5.17 ± 0.14	71.925	<0.001 *
	Female	3.16 ± 0.39	3.28 ± 0.16		
VO_2_max (mL/kg/min)	Male	71.05 ± 7.90	77.0 ± 4.49	17.718	<0.001 *
	Female	60.26 ± 4.20	64.33 ± 3.29		
HRmax (beat/min)	Male	192 ± 9	192 ± 6	0.157	0.698
	Female	192 ± 9	193 ± 11		
AT (L/min)	Male	3.02 ± 0.84	3.29 ± 0.90	4.382	0.028 *
	Female	2.18 ± 0.35	2.28 ± 0.21		
ATHR (beat/min)	Male	155 ± 20	155 ± 15	2.987	0.108
	Female	165 ± 9	170 ± 11		
All-out time (sec)	Male	1038 ± 46.1	1064 ± 49.6	1.427	0.254
	Female	855.2 ± 53.4	874.75 ± 52.6		
Recovery time (sec)	Male	168.8 ± 56.1	102.6 ± 22.9	1.630	0.224
	Female	138 ± 48.8	129.6 ± 56.0		

* p < 0.05; data are presented as the mean ± standard deviation. *p*-values were obtained by analysis of convariance. Abbreviations: BMI, body mass index; VO_2_max, maximal oxygen uptake; HRmax, heart rate maximum; AT, anaerobic threshold; ATHR, anaerobic threshold heart rate.

## Data Availability

The data are not publicly available due to privacy and ethical reasons.
